# Wax-oil lubricants to reduce the shear between skin and PPE

**DOI:** 10.1038/s41598-021-91119-0

**Published:** 2021-06-02

**Authors:** Kian Kun Yap, Manoj Murali, Zhengchu Tan, Xue Zhou, Luli Li, Marc Arthur Masen

**Affiliations:** 1 Department of Mechanical Engineering, Imperial College London, London, UK; 2 Department of Physiology, Anatomy and Genetics, University of Oxford, Oxford, UK; 3 School of Mechanical Engineering, Southwest Jiaotong University, Chengdu, China

**Keywords:** Occupational health, Biomedical engineering, Mechanical engineering

## Abstract

Prolonged use of tight-fitting PPE, e.g., by COVID-19 healthcare workers leads to skin injuries. An important contributor is the shear exerted on the skin due to static friction at the skin-PPE interface. This study aims to develop an optimised wax-oil lubricant that reduces the friction, or shear, in the skin-PPE contact for up to four hours. Lubricants with different wax-oil combinations were prepared using beeswax, paraffin wax, olive oil, and mineral oil. In-vivo friction measurements involving seven participants were conducted by sliding a polydimethylsiloxane ball against the volar forearms to simulate the skin-PPE interface. The maximum static coefficient of friction was measured immediately and four hours after lubricant application. It was found that the coefficient of friction of wax-oil lubricants is mainly governed by the ratio of wax to oil and the thermal stability and morphology of the wax. To maintain long-term lubricity, it is crucial to consider the absorption of oil into the PPE material. The best performing lubricant is a mixture of 20 wt% beeswax, 40 wt% olive oil, and 40 wt% mineral oil, which compared to unlubricated skin, provides 87% (P = 0.0006) and 59% (P = 0.0015) reduction in instantaneous and 4-h coefficient of friction, respectively.

## Introduction

Personal protective equipment (PPE) is an essential tool to fight against viruses, e.g., COVID-19, capable of airborne transmission. Hence, PPE such as goggles, visors, and respirator masks have become critical components for frontline medical staff facing the pandemic. However, the prolonged wearing of facial PPE, especially tight-fitting PPE, can cause various skin injuries, e.g., contact dermatitis, urticaria, skin tears, blisters, and pressure ulcers^1–3^. Skin injuries cause pain and discomfort which reduce an individual’s ability to wear PPE. Worse still, open wounds caused by severe skin injuries may potentially provide a dermal pathway for bacterial and viral infection^4^. Therefore, it is important to devise measures to prevent skin injuries from occurring.

Wearing PPE introduces a combination of normal and shear loads on skin which is a key contributor to pressure-related skin injuries^5–8^. Most studies focus on understanding the distribution of normal loads at skin-PPE interface so that PPE can be redesigned to minimise the normal loads^9–11^. However, this ignores the shear load acting on the skin. Addressing these shear loads, e.g., by applying a lubricant at skin-PPE interface, provides a rapid and practical solution towards alleviating skin injuries during a pandemic^12^. The shear loading is due to static friction at the skin-PPE interface that prevents sliding when the PPE experiences tangential deformation or when there is a tangential load, such as gravity, acting on it. Excessive shear stresses on the skin surface can cause injuries due to significant tissue deformation and cellular distortion^6^. At the same time, shear loading reduces blood perfusion and subsequently decreases the transcutaneous oxygen level in skin^7^. All these aspects weaken skin integrity, making it more prone to injury. Computational simulations by Oomens et al., show elevated shear strains at bony prominences^8^, explaining why facial PPE-related skin injury mainly develops at the bridge of the nose, cheekbones, and forehead^3,13^.

The authors previously investigated various commercially available skin creams and found that lubricants containing natural waxes and oils performed significantly better than other tested products in providing long-lasting lubricity at the skin-PPE interface^12^. In typical skincare products, waxes function as thickeners or viscosity modifiers, whilst oils act as hydrating agents^14^. These two ingredients are also used in commercial anti-chafe balms, which aim to reduce the friction between skin and fabric materials. The use of natural waxes and oils is not limited to skincare applications. Their potential to replace grease to make mechanical systems more environmentally friendly is being investigated^15^. Natural waxes and oils often outperform synthetic or mineral waxes and oils at metal–metal boundary lubricated interfaces due to the high content of fatty acids, such as oleic acid, which form a tribo-film that prevents direct metal–metal adhesion^16,17^. However, at present there is no published work detailing the differences between natural and synthetic wax-oil systems for skin contacts.

The overall aim of this study is to develop a wax-oil lubricant formulation that reduces frictional shear forces at the skin-PPE interface. As part of this, the lubricating properties of both natural and synthetic wax-oil mixtures are experimentally investigated. The objective is to reduce friction to help alleviate PPE-related skin injuries suffered by frontline medics during the COVID-19 pandemic.

## Materials and methods

The study involved human participants and was approved by the Imperial College London Science, Engineering and Technology Research Ethics Committee (SETREC), reference 20IC6330. All experiments were performed in accordance with relevant guidelines and regulations. Written informed consent was obtained from all participants and/or their legal guardians before testing.

### Skin-PPE contact interface

**Figure 1:**
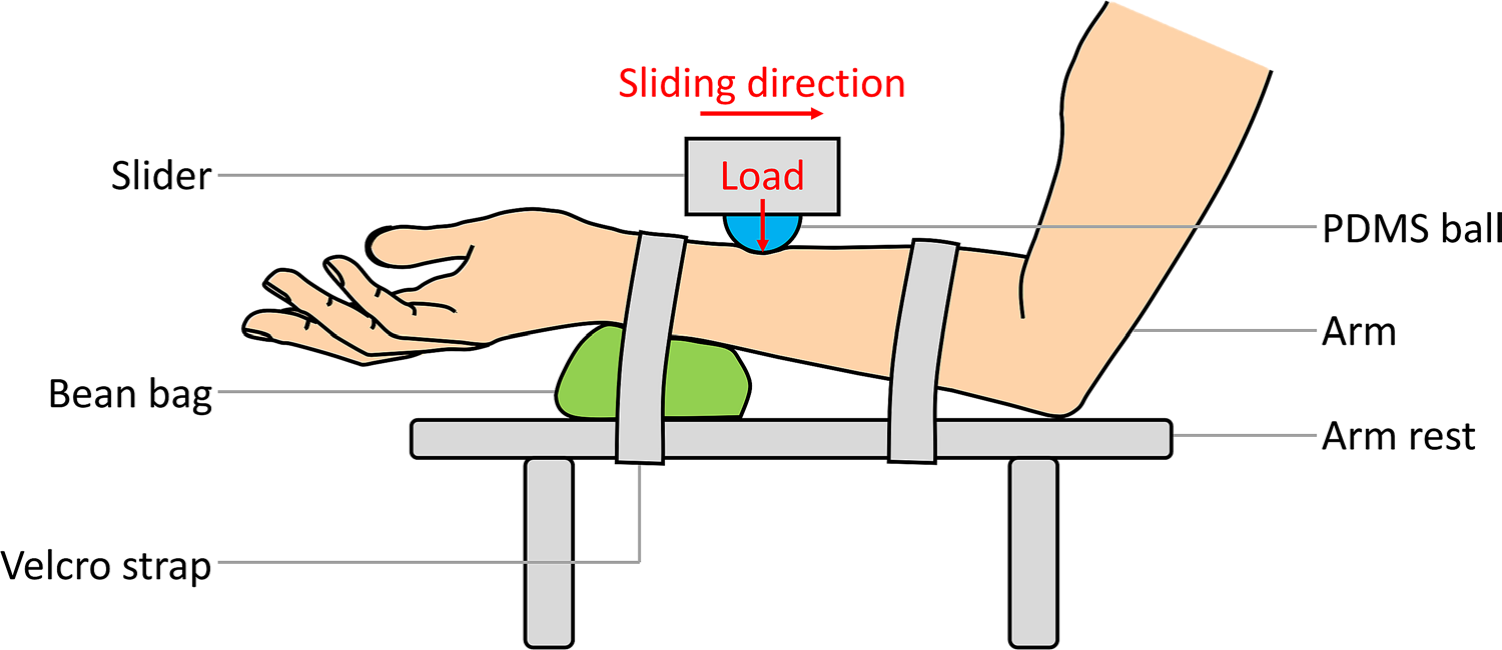
Schematic of the in-vivo tribometer setup (the image was created by Microsoft PowerPoint 2016).

In-vivo friction tests were performed to simulate the facial skin-PPE interface using a Universal Mechanical Tester (UMT, Bruker Corporation, Germany). A schematic of the setup is shown in Fig. 1. The device measures the friction in the contact between a moving upper specimen and a stationary lower specimen. A polydimethylsiloxane (PDMS) specimen was chosen to represent the PPE material. PDMS is commonly used as the material that interfaces with skin in tight-fitting PPE. As a common practice in skin tribology studies, facial skin was simulated by performing tests on the volar forearm^18–22^. Hendriks et al. showed there is no significant difference in friction values measured on the forearm and the cheek^22^. The contact pressures for ventilator masks range between 8 and 30 kPa^23^. Kuilenburg suggested an effective Young’s modulus of 50 kPa for skin^24^. Hence, an 18 mm PDMS ball (Silex Limited, UK) with a Young’s modulus of 1.12 MPa was used to obtain a mean Hertzian contact pressure of 14 kPa when a 1 N load was applied.

### Lubricant preparation

Various lubricants were prepared using four ingredients that are safe to apply on human skin: refined yellow beeswax (melting point 62 to 65 °C, Sigma Aldrich UK, 243248), paraffin wax (melting point 50 to 59 °C, Sigma Aldrich UK, 18634), olive oil (viscosity 44.3 mPa·s, density 0.85 g/cm^3^, Sigma Aldrich UK, 75343), and neat light mineral oil (viscosity 17.2 mPa·s, density = 0.84 g/cm^3^, Sigma Aldrich UK, M5310). The chemical constituents of these ingredients are listed in Table 1. Melting points were measured by observing the temperature at which the waxes melted completely. Viscosities and densities were measured using a viscometer (SVM 3000, Anton Paar, Austria) at 34 °C, as this is the surface temperature of human skin^25,26^. The reason for selecting these ingredients is because they are the common base ingredients used in commercial wax-oil skin creams. Beeswax (BW) and olive oil (OO) represent ingredients for a natural system whilst paraffin wax (PW) and mineral oil (MO) represent that of a synthetic system.

**Table 1:** General chemical constituents of ingredients used in wax-oil lubricants.

Ingredients	Chemical constituents	References
Beeswax (BW)	50–72% esters with carbon atoms ranging from C40–C48, 12–16% hydrocarbons of C27–C33, 12–14% free fatty acids of C24–C32, and 1% fatty alcohols of C28–C35	Fratini, et al.^27^
Paraffin Wax (PW)	Saturated hydrocarbons (alkanes) with chain length ranging from C18–C36	Rehan, et al.^28^
Olive Oil (OO)	Olive oil composition: 99% triglycerides and 1% free fatty acids, mono and diglycerides, and lipids Fatty acid and triglycerides composition: 55–83% oleic acid (C18:1), 7.5–20% palmitic acid (C16:0), 3.5–21% linoleic acid (C18:2), and others with carbon atoms ranging from C14–C24 (mainly C16 and C18)	Boskou, et al.^29^ and Tsimidou, et al.^30^
Mineral Oil (MO)—Light	Saturated hydrocarbons (alkanes) with chain length ranging from C15–C25	Nash, et al.^31^ and Rawlings, et al.^32^

To prepare a wax-oil lubricant, wax was heated to 100 °C until it melted completely. Oil was then added to make a total of 20 g of lubricant at a desired wax-oil ratio by weight. A drop of vitamin E (Sigma Aldrich UK, 90669), which acts as an antioxidant, was added to the mixture to minimise the oxidation of the lubricant^14^. The mixture was kept at 100 °C for another 15 min and stirred using a magnetic stirrer to achieve homogeneity after which it was left to cool to room temperature. Four types of lubricants were prepared:

- paraffin wax-olive oil (PWOO)
- paraffin wax-mineral oil (PWMO)
- beeswax-olive oil (BWOO)
- beeswax-mineral oil (BWMO)

Initial tests were performed for each type of lubricant using mixtures with wax concentrations of 0, 10, 20, and 30 wt%, and subsequently the range of concentrations was further refined at 5 wt% intervals for mixtures that provided low friction results. As a naming convention for the various mixtures, the weight percent of each component is listed in front of the specific component, e.g., 20BW80OO represents a 20 wt% beeswax and 80 wt% olive oil mixture.

To characterise the various prepared lubricants, their microstructures were studied. The samples were placed on a temperature-controlled glass slide, covered with a coverslip, and observed under a brightfield microscope (Axioskop, Zeiss, Germany) at 50X magnification. Both micrographs at 21 °C (room temperature) and 34 °C (skin surface temperature) were taken to examine the effect of temperature on the properties of the lubricants. All raw micrographs were converted to grayscale and their brightness and contrast were adjusted to similar levels to enable comparison.

### Experimental protocol

#### Instantaneous test (friction measured immediately after lubricant application)

Before commencing each sliding test, a new PDMS ball was installed on the slider and wiped with isopropanol. The right volar forearm of the participant was washed using water and non-soap hand wash (Simple Pure Hand Wash, Unilever, UK) and dried using paper towels. No hair removal procedure was performed. The corners of a 3 × 5 cm^2^ area were marked with a permanent marker on the forearm, starting 4 cm proximally from the wrist, and 120 mg of lubricant was applied to this area using the index finger of the other hand. This resulted in a coverage of approximately 8 mg/cm^2^, which was deemed to be a generous amount but not excessive. A small amount of lubricant will remain on the index finger after application and this amount might vary slightly in each repeat. However, the effect of this on the obtained results will be minimal. In addition, the method replicates the likely action of how a user would apply a lubricant on skin.

During a sliding test, the forearm with the lubricated area was placed on an arm rest in a relaxed position underneath the PDMS ball as shown in Fig. 1. The surface of the forearm was levelled by adjusting the position of the bean bag under the arm in conjunction with a spirit level on the arm. The position of the arm was then secured using Velcro straps. The test started by moving the PDMS ball towards the surface of the forearm, until the actively-controlled 1 N load was exerted. Then, the PDMS ball slid for 20 mm proximally across the forearm at a speed of 1 mm/s. After sliding, the PMDS ball was raised and moved to its original position.

#### Four-hour test (friction measured four hours after lubricant application)

The developed lubricants should be able to maintain their lubricity for the duration that the PPE is worn. The recommended continuous wear time for most tight-fitting respirators is less than three hours^33,34^. Therefore, in addition to the instantaneous test, a friction test was also conducted four hours after application, on selected lubricants that showed good performance in the instantaneous tests, to evaluate their performance over an extended duration. In this test, the skin site with applied lubricant was covered by a PDMS sheet which was held in place using fabric plasters and the participants were allowed to carry out their daily routine for four hours, after which the friction test was performed.

#### Subjects

To ensure the reproducibility of results, all initial scoping tests were performed on three volunteers. The follow-up instantaneous and 4-h tests using the various selected lubricants from the scoping tests were repeated on seven and four volunteers respectively. The volunteers consisted of four males and three females with ages ranging from 23 to 28 years. The narrow age range was due to COVID-19 restrictions in place during the experimental programme. However, literature shows that there is no significant difference in the skin friction for different age groups^20,35,36^.

#### Data acquisition and data processing

**Figure 2:**
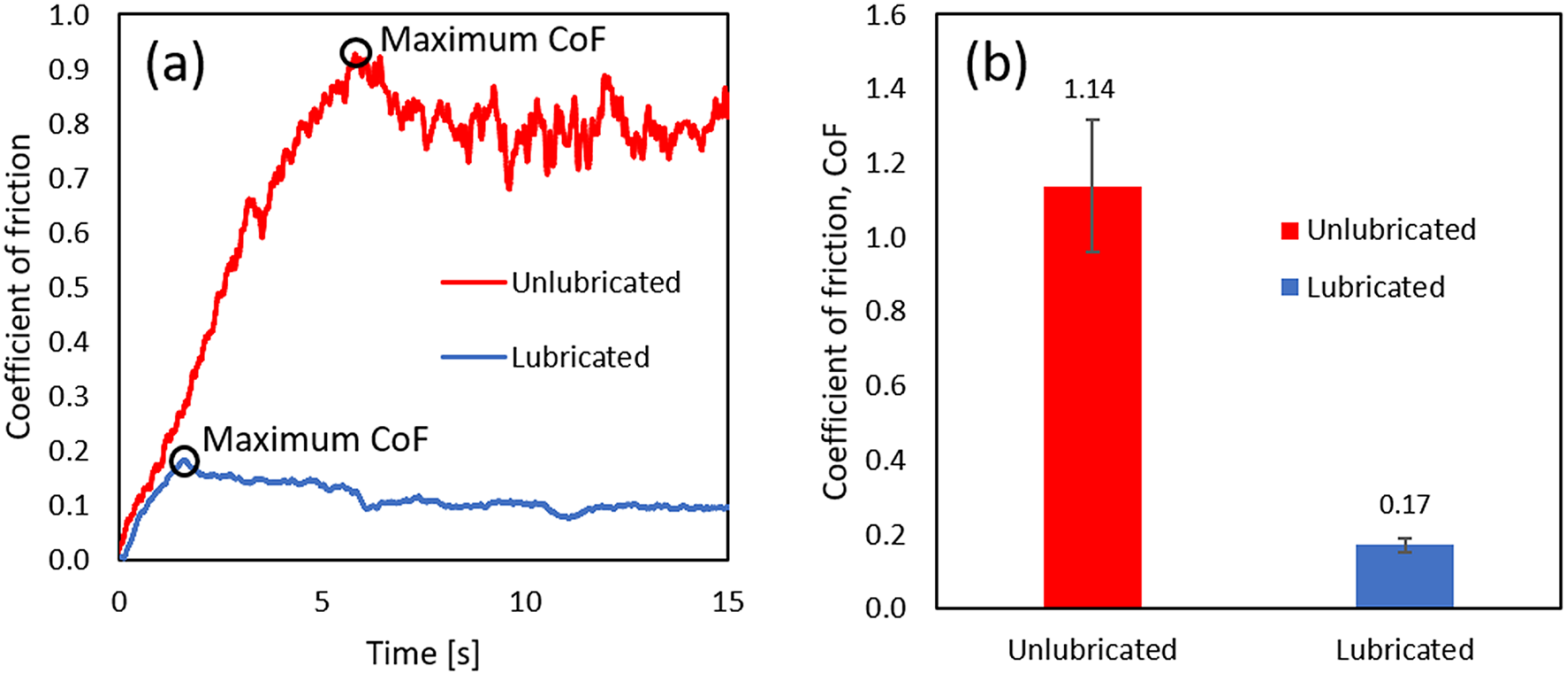
(**a**) Typical friction curves obtained from an individual in-vivo sliding test and (**b**) the average maximum static CoF experienced by the entire cohort and its standard deviation.

The UMT recorded the normal and friction forces during sliding at a sampling rate of 2000 Hz. Figure 2a shows two typical coefficient of friction (CoF) curves obtained, on unlubricated and lubricated skin. The maximum CoF during the initial stage of sliding, which represents the transition from static to dynamic friction, was used to characterise the severity of shear. This maximum static friction relates to the maximum shear stresses that can occur at the skin-PPE interface. The average maximum static CoF was calculated from the maximum values obtained on the various volunteers and the standard deviation was plotted as the error bars as shown in Fig. 2b. The values above the bars represent the average maximum static CoF. Later in the discussion, p-values calculated from the dependent two-tailed t-test for paired samples will be used to evaluate the statistical significance of the difference in CoF between two compared lubricants. A p-value which is smaller than or equal to 0.05 (P ≤ 0.05) indicates that the difference is statistically significant at a 95% confidence interval.

## Results

Note that despite this study being a continuation of previous work^12^, direct comparison of friction values should be avoided as the equipment, the sliding parameters, and the test protocols used are not the same.

### Instantaneous test (friction measured immediately after lubricant application)

**Figure 3:**
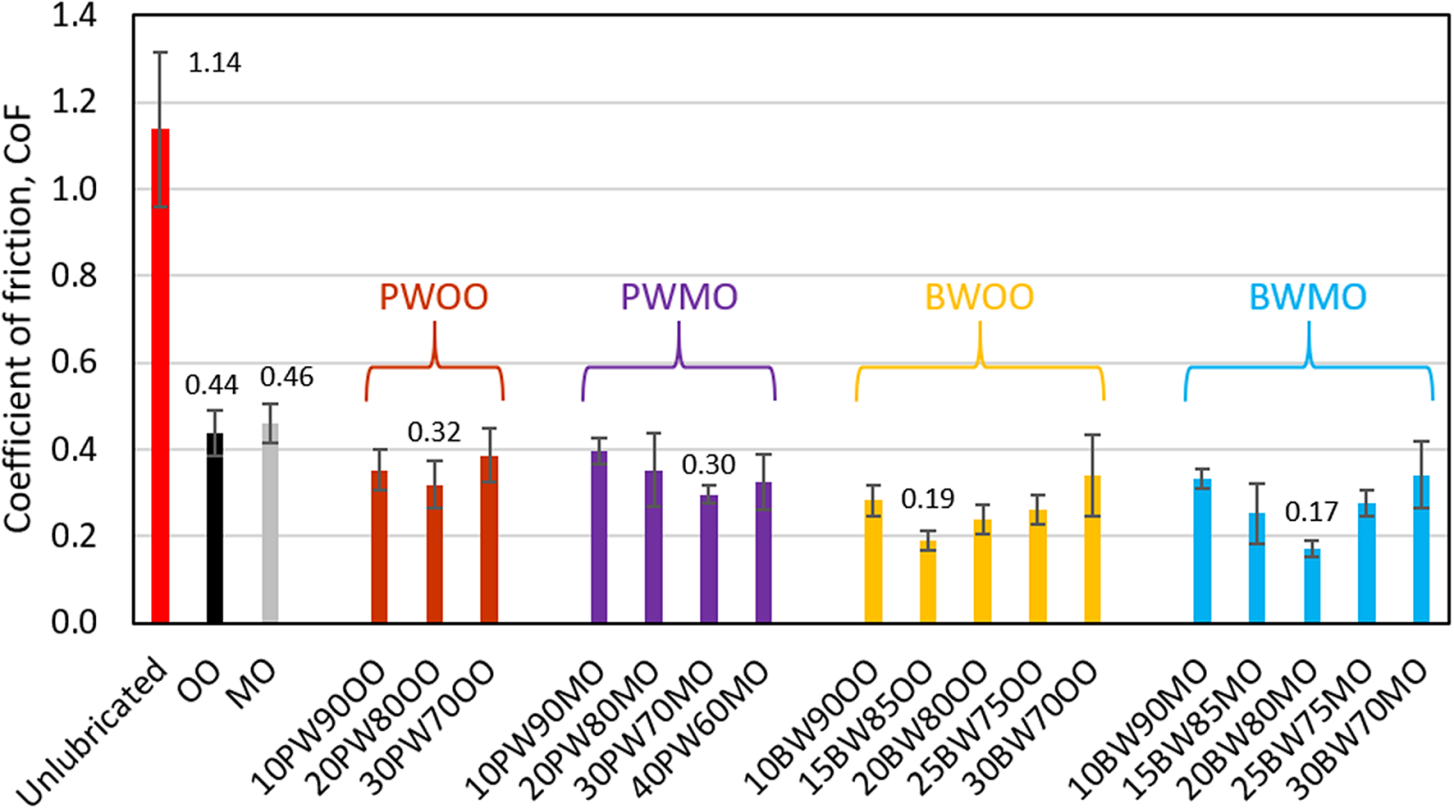
Overview of instantaneous static CoF of various lubricants with unlubricated skin as a baseline for comparison. BW stands for beeswax, PW is paraffin wax, OO is olive oil, and MO is mineral oil. As a naming convention for the various mixtures, the weight percent of each component is listed in front of the specific component, e.g., 20BW80MO represents a 20 wt% beeswax and 80 wt% mineral oil mixture.

Figure 3 shows the CoF measured for the various lubricants in the instantaneous tests. The leftmost bar, in red, represents the CoF of unlubricated skin, which is used as a baseline to compare the efficacy of lubricants. Additionally, values for pure olive oil and pure mineral oil (i.e., 0 wt% wax) are shown in black and grey, respectively. In general, all lubricants provide at least a 59% reduction in friction in the instantaneous tests as compared to unlubricated skin (P = 0.0202). The friction obtained for each of the wax-oil lubricant displays a strong dependence on the wax concentration. The various wax-oil lubricants with the lowest friction perform at least 27% better than the corresponding pure mineral oil and olive oil lubricants (P = 0.0488). The CoF measured for the beeswax-based lubricants is at least 36% lower than the paraffin wax-based lubricants (P = 0.0425). The lowest CoF is found in the beeswax-mineral oil mixture, which is 85% lower than unlubricated skin (P < 0.0001) and 9% lower than the lowest CoF value obtained for beeswax-olive oil (P = 0.0035).

### Four-hour test (friction measured four hours after lubricant application)

**Figure 4:**
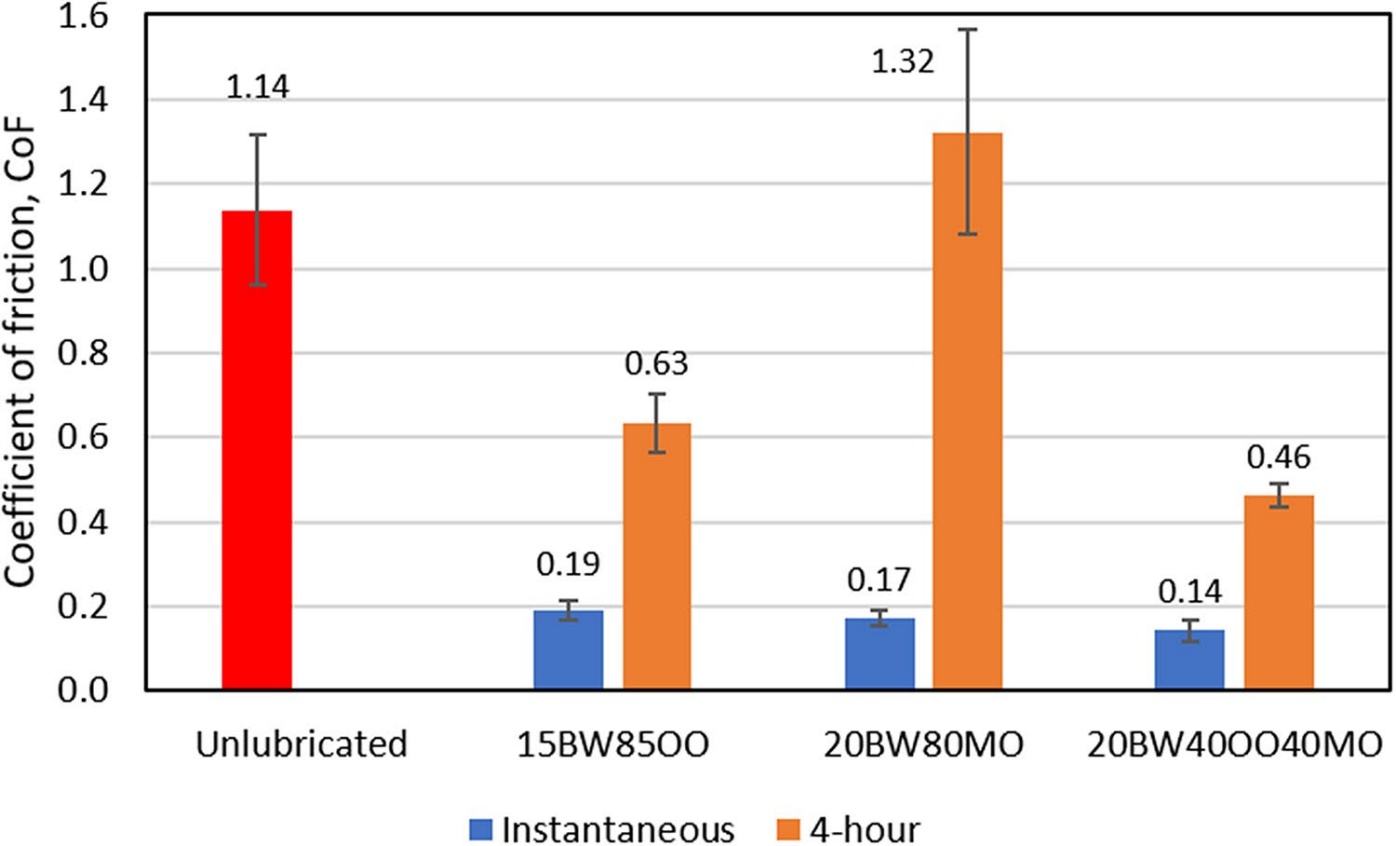
Instantaneous and 4-h static CoF of selected lubricants. BW stands for beeswax, PW is paraffin wax, OO is olive oil, and MO is mineral oil. As a naming convention for the various mixtures, the weight percent of each component is listed in front of the specific component, e.g., 20BW80MO represents a 20 wt% beeswax and 80 wt% mineral oil mixture.

The two best performers from the instantaneous test, namely 15BW85OO and 20BW80MO, were selected for the 4-h test. Additionally, based on the gained insights from the wax microstructure and the absorption of oil into PDMS, which will be discussed later in the paper, a third wax-oil lubricant combination variant that contains 20 wt% beeswax, 40 wt% olive oil, and 40 wt% mineral oil (20BW40OO40MO) was introduced. Figure 4 shows both the instantaneous and 4-h CoF for these selected lubricants. In general, the CoF four hours after application is increased compared to immediately after application. However, the beeswax-olive oil mixture still results in a 44% reduction of the CoF as compared to that of unlubricated skin (P = 0.0030). In contrast, the CoF of beeswax-mineral oil four hours after application is 16% higher than for unlubricated skin (P = 0.0403). Out of all tested lubricants, the beeswax-olive oil-mineral oil mixture shows the lowest friction, providing 87% (P = 0.0006) and 59% (P = 0.0015) reduction in instantaneous and 4-h CoF compared to the unlubricated skin. Its instantaneous CoF is 16% lower than that of beeswax-mineral oil (P = 0.0133) and its 4-h CoF is 27% lower than that of beeswax-olive oil (P = 0.0386).

### Microstructures of wax-oil lubricants

**Figure 5:**
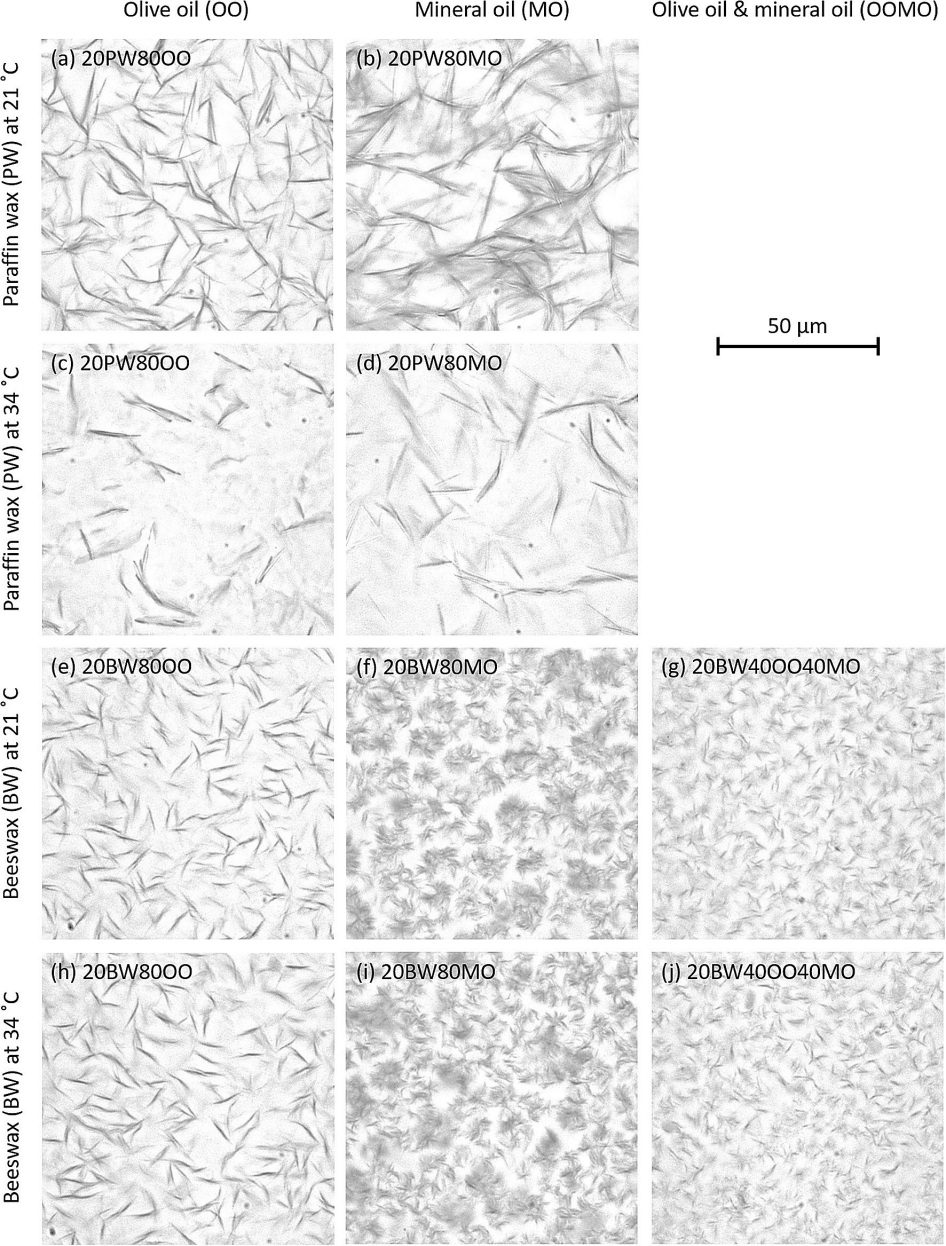
Microstructures of various wax-oil lubricants at 20 wt% wax and 80 wt% oil at 21 °C (room temperature) and 34 °C (skin surface temperature). As a naming convention for the various mixtures, the weight percent of each component is listed in front of the specific component, e.g., 20BW80MO represents a 20 wt% beeswax and 80 wt% mineral oil mixture.

The obtained friction results provide an overview of the functional performance of wax-oil lubricants. However, to elucidate the mechanisms governing the friction response, it is crucial to understand the composition of the wax-oil lubricants. Figure 5 shows the microstructures of various 20 wt% wax and 80 wt% oil lubricants at 21 °C (room temperature) and 34 °C (skin surface temperature). As shown in these micrographs, a wax-oil mixture is generally a colloidal system in which the wax crystals disperse uniformly in the oil matrix. Figure 5a,b show that at 21 °C, paraffin waxes have a needle-like structure with a characteristic size in the order of tens of micrometres. However, both the amount and the size of the crystals reduce sharply at 34 °C, as shown in Fig. 5c,d. Beeswax displays a distinctive crystal morphology in the different base oils, as shown in Fig. 5e–g, with no significant change in the morphology when the temperature is increased to 34 °C as shown in Fig. 5h–j. In olive oil, Fig. 5h, a large amount of short needle-like crystals is found, whilst in mineral oil, Fig. 5i, beeswax forms conglomerates of highly branched dendritic crystals and these islands, with a size of eight to ten microns, appear separated. When beeswax is mixed with both olive oil and mineral oil, as shown in Fig. 5j, both needle-like and dendritic crystals are found, and the characteristic length scale of the crystals is much reduced.

## Discussion

### Instantaneous test (friction measured immediately after lubricant application)

The total shear force acting on the skin, *F*_*f*_ is the combination of forces due to the viscoelastic deformation of the materials in contact, *F*_*bulk*_ and forces due to the breaking of intermolecular bonds at the contact interface, *F*_*int*_ as shown in Eq. (1)^37,38^. The contribution of bulk deformation to the friction is often assumed to be negligible^22^:

1 $${F}_{f}={F}_{bulk}+{F}_{int}\approx {F}_{int}$$

The interfacial friction force, *F*_*int*_ is the product of the contact area, *A* and the shear strength of the contact interface, *τ*_*int*_. This results in Eq. (2) which relates *τ*_*int*_ to *F*_*f*_:

2 $${F}_{f}\approx {F}_{int}={\tau }_{int}\cdot A$$

The introduction of a lubricant to the contact provides a thin, low-shear interface. This reduces *τ*_*int*_ and therefore decreases the friction as observed for all lubricated conditions in Fig. 3.

The wax-containing lubricants in our study generally perform better than pure oil lubricants as wax crystals function as thickeners which keep the oil in the contact, preventing outflow of oils from the interface upon loading^39^. This reduces the amount of direct skin-PDMS contact and therefore reduces friction. When the wax concentration is too low the wax crystal fails to retain sufficient oil to lubricate the contact interface, whilst when the wax concentration is too high, the wax-oil mixture becomes too thick and creates excessive viscous drag. The reduced friction observed for beeswax-based lubricants compared to paraffin wax-based lubricants can be related to the higher melting point of beeswax; the high degree of paraffin wax decrystallisation at 34 °C as shown in Fig. 5c,d will lead to a severe reduction of the thickening effect in the lubricants and thus an increase in the outflow of the lubricant from the contact interface.

Beeswax-mineral oil shows a lower instantaneous CoF than the beeswax-olive oil mixture. This contrasts with other results obtained for these oils, where similar values of the CoF were obtained for the two base oils (P = 0.6720) as well as for the two paraffin wax-based mixtures (P = 0.6605). This implies that for beeswax-based lubricants the friction mechanism is not governed by the properties of the base oils, but by the properties of the resulting wax-oil mixtures, which in this case is the morphology of wax crystals. As stated in Table 1, beeswaxes contain a large amount of ester groups (O=C–O) in the esters and carboxylic groups (COOH) in the fatty acids. These polar functional groups make the beeswax to have strong chemical affinity for olive oil, which also contains ester groups (O=C–O) and carboxylic groups (COOH). Beeswaxes show weak chemical affinity for mineral oils, which comprise 100% non-polar hydrocarbons. For this reason, the polar groups in beeswaxes prefer adhering to each other rather than integrating with the mineral oil, and instead of forming a needle-like shape in a mineral base oil, the beeswax crystals tend to form spherical highly branched dendritic structures to minimise the surface energy of the system in a steady state. The weak chemical affinity between beeswaxes and mineral oils is further substantiated by the significant bleeding of oil observed for beeswax-mineral oil mixtures several days after preparation, which is identified by small pools of oil at regions where the surface of the mixture is not flat. It is hypothesised that the spherical crystals in beeswax-mineral oil mixtures act as effective rollers in the skin-PDMS interface, resulting in lower friction than beeswax-olive oil mixtures, which have a needle-like crystal structure.

During the crystallisation process, beeswax will form needle-like structures when it is surrounded by the polar olive oil and dendritic structures when it is surrounded by the non-polar mineral oil. When using both oils, a combined structure that comprises both needle-like and dendritic crystals can be obtained, as shown in Fig. 5j. The resulting crystals have a smaller size, as needle-growth is halted when the nuclei encounter non-polar mineral oil molecules, whilst dendrite growth is disrupted by the polar olive oil molecules. An apparent manifestation of this reduced crystal size is the reduced instantaneous friction observed for a beeswax-olive oil-mineral oil mixture compared to beeswax-olive oil and beeswax-mineral oil mixtures. It is hypothesised that the reduced crystal size facilitates the evolution of shear planes in the wax, resulting in relatively low friction. The crystal shape and size could be design parameters in optimising wax-oil lubricants further.

### Four-hour test (friction measured four hours after lubricant application)

The CoF measured four hours after application is higher than the instantaneous CoF, with the beeswax-mineral oil giving higher friction than the beeswax-olive oil mixture as shown in Fig. 4. Visual inspection showed that the beeswax-mineral oil mixture had dried after four hours, resulting in a thin solid film on the skin surface, which caused a CoF that was higher than measured for unlubricated skin. In contrast, for the beeswax-olive oil mixture, although the lubricant remaining four hours after application had clearly reduced, there was still an observable thin layer of liquid lubricant covering the surface that contributed to a reduced shear strength at the interface*.* The frictional performance of the lubricants four hours after application can be directly related to the remaining quantity of lubricant on the surface, which is dependent on the absorption of the lubricants into the skin and/or the PPE material.

#### Oil absorption of PDMS

**Figure 6:**
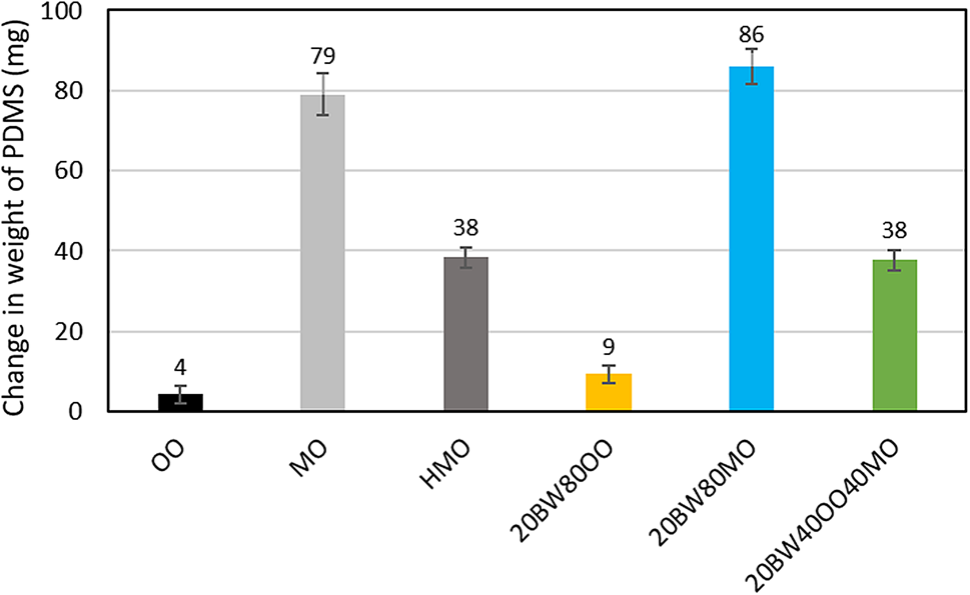
Change in weight of PDMS four hours after the application of lubricants. BW stands for beeswax, PW is paraffin wax, OO is olive oil, MO is mineral oil (light), and HMO is heavy mineral oil. As a naming convention for the various mixtures, the weight percent of each component is listed in front of the specific component, e.g., 20BW80MO represents a 20 wt% beeswax and 80 wt% mineral oil mixture.

To better understand the lubricant absorption into the PDMS specimens, an absorption experiment was conducted. The absorption of oils into the PDMS at skin surface temperature was simulated by keeping PDMS sheets with the desired lubricants in a temperature-controlled environment at 34 °C for a period of four hours. The weight of each PDMS sheet, as well as the total weight of the PDMS with lubricant was measured before and after the test. No change in total weight was observed after four hours for any of the lubricants tested, indicating that the lubricants did not evaporate. After the 4-h holding period, the remaining lubricant was wiped from the surface using isopropanol, after which the weight of the PDMS sheet was measured. Any change in PDMS weight is therefore attributed to absorption. Each absorption test was performed three times to ensure the reproducibility of results. The change in weight of PDMS due to different lubricants, which represents the absorption of oil into PDMS, is shown in Fig. 6.

The absorption of mineral oil into the PDMS is 18 times higher than olive oil. This result is also observed for the beeswax-based lubricants, the absorption for the beeswax-mineral oil is 9 times higher than the beeswax-olive oil mixture. There are two factors which can contribute to this difference, namely the functional groups of oil molecules and the viscosity of the oils. The mineral oil and the olive oil applied in this study have different viscosities, and a higher viscosity signifies larger molecules which would not absorb as easily into the PDMS. Repeating the absorption experiment using heavy mineral oil (HMO, viscosity of 44.6 mPa·s and density of 0.85 g/cm^3^ at 34 °C, Sigma Aldrich UK, 330,760) with a similar viscosity as olive oil resulted in absorption that was approximately half of the original light mineral oil, but still 9 times higher than that of olive oil. This suggests that the chemical interaction between the various oils and the PDMS also plays a vital role. PDMS is a highly non-polar material due to its methyl groups (CH_3_)^40^. Mineral oils applied are mainly composed of saturated hydrocarbons, such as alkanes and cycloalkanes, which are also non-polar and thus have a strong affinity for PDMS. For the case of olive oil, the ester group (O=C–O) in triglycerides and the carboxylic group (COOH) in fatty acids are polar, hence the reduced absorption. Increased oil absorption contributes to an increase of the CoF four hours after application because the resulting wax concentration of the wax-oil lubricant increases when oil is drawn from the mixture into the PDMS, thereby increasing viscous drag.

However, the oil absorption of PDMS is not the sole contributor to frictional behaviour. In the case of the beeswax-olive oil-mineral oil mixture, the combination of olive oil and mineral oil means that the absorption lies between the values for beeswax-olive oil and beeswax-mineral oil as shown in Fig. 6. For this reason, the 4-h CoF of beeswax-olive oil-mineral oil is lower than that of beeswax-mineral oil as shown in Fig. 4. Despite a higher absorption, the 4-h CoF of beeswax-olive oil-mineral oil is lower than that of beeswax-olive oil. This suggests that the 4-h CoF is not only governed by the oil absorption of PDMS but also the morphology of wax crystals as discussed in previous section.

#### Underlying mechanisms of skin friction

As stated in Eq. (1), the total friction is a combination of interfacial and bulk material effects, with the bulk effects often assumed to be negligibly small. However, the softening of skin due to hydration and/or occlusion as well as absorption of substances into the epidermis may affect the bulk mechanical characteristics of the skin. Two further comparative experiments were performed on the beeswax-olive oil-mineral oil mixture to investigate these aspects in detail: 1. the ‘4-h no-PDMS-contact’ test which is similar to the original 4-h sliding test but with a custom cover being used that prevents contact of the PDMS with the lubricated site of the arm during the 4-h waiting time, thus preventing the absorption of lubricant into the PDMS. These results would eliminate the effects of oil absorption into the PPE material. 2. the ‘4-h relubricated’ test which is also similar to the original 4-h test except the skin surface is relubricated just before performing the sliding test. Any differences between these results and the instantaneous tests would indicate an effect of the lubricant on the skin properties.

**Figure 7:**
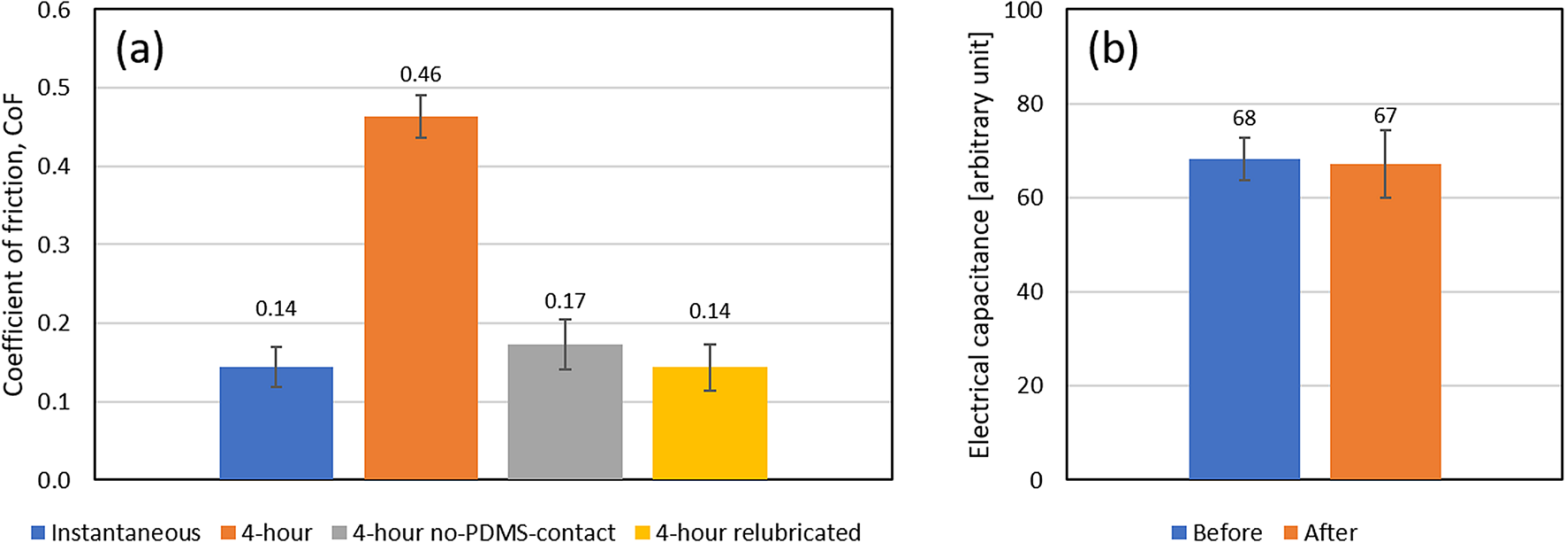
(**a**) Static CoF of 20BW40OO40MO and (**b**) electrical capacitance of stratum corneum before and four hours after the application of 20BW40OO40MO. 20BW40OO40MO represents a lubricant that contains 20 wt% beeswax, 40 wt% olive oil, and 40 wt% mineral oil.

As shown in Fig. 7a, the CoF of ‘4-h no-PDMS-contact’ test is 17% higher than the instantaneous test (P = 0.0516). This can be attributed to (i) the softening of skin due to the hydration caused by the occlusive property of wax-oil lubricant, (ii) the softening of skin due to the oil absorption itself, and/or (iii) the reduction of oil on the skin surface which increases the resulting wax concentration in the lubricant. As shown in Fig. 7b, the electrical capacitance which represents the hydration level of stratum corneum was measured before and four hours after the application of lubricant using a corneometer (CM 825, Courage + Khazaka Electronic, Germany). Before the hydration measurement, any lubricant was removed from the skin using a paper towel. Since there is no significant change in the hydration level of the stratum corneum four hours after lubricant application (P = 0.5477), the first hypothesis can be opted out. There is also no significant change between the CoF of ‘4-h relubricated’ test and instantaneous test (P = 0.9552). This suggests that there is no significant change in the mechanical properties of the skin that could affect its friction and therefore the second hypothesis can be eliminated. Hence, it is concluded that the increase in ‘4-h no-PDMS-contact’ CoF is due to a reduction in lubricant on the skin surface due to oil absorption into the skin. However, the fact that the CoF of the original 4-h test is much higher than the ‘4-h no-PDMS-contact’ test as shown in Fig. 7a indicates that the effect of oil absorption into the PPE-material is much more dominant than that into the skin.

## Conclusion

Of the investigated lubricants, the 20 wt% beeswax, 40 wt% olive oil, and 40 wt% mineral oil mixture gives the best long-term (four hours) lubricity at the skin-PPE interface. The minimum CoF can be achieved by having an optimal wax-oil ratio to balance between the oil retaining capability and the viscous drag of the lubricant. The higher thermal stability of beeswax makes it a better thickener than paraffin wax. The morphology of wax crystals, which is determined by the chemical affinity between the wax and the oil in the mixtures, is also crucial in controlling the CoF. A fine spherical dendritic wax structure in the beeswax-olive oil-mineral oil mixture is preferable. The long-term lubricity of a wax-oil mixture is mainly limited by the absorption of oil into the PPE material. A cost-effective solution would be to develop lubricants with a weak affinity for the PPE materials instead of redesigning the PPE. In the investigated case, adding olive oil, which has a much lower absorption into the PPE, into the mineral oil results in a lubricant with a moderate absorption into the PPE, whilst maintaining a favourable wax crystal structure to ensure low CoF in the longer term. Such an optimised lubricant formulation could reduce PPE-related skin injuries among COVID-19 healthcare workers.

## Data Availability

The data that support the findings of this study are available from the Imperial College Institutional Research Data Repository, https://doi.org/10.14469/hpc/8076.
